# Surface Topography and Pushout Bond Strength of Glass Fiber Posts with Different Surface Treatments to Root Dentin Following Cementation by Precuring and Co-Curing Methods: Short title: Surface Topography and Pushout Bond Strength of Glass Fiber Posts to Root Dentin Following Cementation

**DOI:** 10.31661/gmj.v15i.3865

**Published:** 2026-05-02

**Authors:** Azita Kaviani, Pardis Khoshnood, Faramarz Zakavi

**Affiliations:** ^1^ Department of Restorative Dentistry, School of Dentistry, Ahvaz Jundishapur University of Medical Sciences, Ahvaz, Iran

**Keywords:** Surface Properties, Fiberglass, Cementation, Light-Curing of Dental Adhesives

## Abstract

**Background:**

This study assessed the surface topography and pushout bond strength (PBS) of glass fiber posts (GFPs) with different surface treatments to root dentin following cementation by precuring and co-curing methods.

**Materials and Methods:**

In this in vitro study, 80 extracted single-rooted premolars were decoronated and randomly assigned to 4 groups (n=20) for surface treatment of GFPs with 20% H2O2, Er,Cr:YSGG laser (2780 nm, 90 mJ, 4.5 W, 50 Hz, 60 seconds) and sandblasting (50 µm aluminum oxide particles, 2.8 bar pressure, 10 mm distance, 20 seconds). No surface treatment was performed in group 4. Each group was randomly divided into two subgroups (n=10) for cementation of GFPs with precuring and co-curing methods. The PBS was measured, the mode of failure was determined, and the surface topography of the posts was assessed under a scanning electron microscope (SEM). Data were analyzed by ANOVA, Tukey, and Dunnett tests (alpha=0.05).

**Results:**

The mean PBS of H2O2-treated GFPs was significantly higher in co-curing than precuring method (P=0.04). No significant difference was found between the precuring and co-curing in PBS of GFPs with other surface treatments (P0.05). The mean PBS of sandblasted, laser-treated, and H2O2-treated GFPs was not significantly different in precuring method. In co-curing cementation, sandblasting significantly decreased the PBS (P=0.04) while laser and H2O2 surface treatments had no significant effect on PBS (P0.05).

**Conclusion:**

The co-curing cementation method yielded a significantly higher PBS than the precuring method in H2O2-treated GFPs. Sandblasting significantly decreased the PBS of GFPs cemented by the co-curing method.

## Introduction

Excessive loss of the tooth structure due to caries, trauma, or tooth preparation often necessitates root canal treatment, which increases the susceptibility to tooth fracture especially in teeth with mesio-occluso-distal cavities [1]. To elaborate, a 46% loss in tooth rigidity has been reported following the loss of one marginal ridge; this value increases to 63% following the loss of both marginal ridges [2].

Endodontically treated teeth can be restored by different methods, with the understanding that preservation of tooth structure and adhesion of restorative materials to dental substrate determine the success of treatment [1]. Intracanal posts are required to support the core and allow for better stress distribution in teeth that have lost over 50% of their structure [3]. Consequently, the success rate is higher for teeth with a post and core restoration [1].

Fiber posts have a modulus of elasticity (16-40 GPa) close to that of dentin (18.6 GPa), enabling uniform stress distribution along the root surface [4]. Furthermore, they have optimal esthetics for use under ceramic restorations. Additionally, they can bond to composite resin cores, have optimal biocompatibility and corrosion resistance, and require a short treatment time [5]. They are also characterized by high fatigue resistance and flexibility, which decrease the risk of root fracture and mechanical failure (which would necessitate tooth extraction) [6]. Glass fiber posts (GFPs) are composed of reinforced glass fibers embedded in epoxy polymethyl methacrylate or urethane dimethacrylate resin, both having the same flexural strength. Importantly, the retention of GFPs in the root canal system depends on proper adhesion of resin cement and intra-radicular dentin, as well as the adhesion between the cement and post surface. In other words, the retention of GFPs depends on chemical and micro-mechanical interactions between the post, dentin, and resin cement. Failure of GFPs often occurs due to debonding at the cement-dentin or cement-post adhesive interface as a result of bonding deficiencies. Thus, several mechanical and chemical post surface treatments have been proposed to enhance the bond strength of fiber posts to root dentin [7], such as grit-blasting, airborne particle abrasion, silanization, etching, and laser irradiation [5][8][9][10][11][12][13]. The goal of the suggested methods is to roughen the surface [11][14][15] or chemically treat the surface of glass fiber reinforced resin composite posts to increase mechanical cross-linking of the resin cement matrix [16][17]. Surface roughening can be performed in macro-, micro-, or nanoscale, depending on the surface treatment parameters [11][16][17]. Moreover, a combination of physical and chemical methods may be used to improve the bond strength of glass fiber reinforced resin composite posts to resin cements [8][11][16]. For example, a previous study demonstrated that fiber post surface treatment by sandblasting with 30 µm aluminum oxide particles with 2.5 bar pressure from 2 cm distance for 10 seconds roughened the surface and increased the bond strength [7]. Similarly, another study showed an improvement in micro-pushout bond strength (PBS) of GFPs treated by hydrogen peroxide (H₂O₂) and 1 W and 1.5 W erbium, chromium, yttrium, scandium, gallium garnet (Er,Cr:YSGG) laser [18].

The cementation technique also directly affects the retention of posts and plays an important role in treatment success. Specifically, the luting agents can be categorized into self-adhesive, and total-etch and self-etch (SE) bonding systems, depending on their adhesion mechanism. The SE bonding systems are extensively used for cementation of fiber posts. Generally, two methods are available for the application of SE bonding systems with a resin cement. In the first approach, known as the precuring method, the bonding layer is light-polymerized before the application of cement. In the second approach, referred to as the co-curing method, the bonding layer is cured simultaneously with the cement [19]. No specific protocol exists regarding the selection of precuring or co-curing method. Nonetheless, the precuring method for cementation of fiber posts may decrease the bond strength due to the thickness of the bonding layer and its adverse effect on complete seating of the post in the canal space. Thus, controversy exists regarding the selection of precuring or co-curing approach for cementation of GFPs.

Aside from the bond strength, topographic changes of the surface following post surface treatments may also decrease the PBS of GFPs. In particular, the risk of degradation of resin structure during the surface treatment process is important and should be taken into account. For instance, broken fibers and cracks noticed on scanning electron microscopic (SEM) images have been considered as the possible causes of compromised PBS of GFPs [20].

Considering the availability of various surface treatments and different cementation techniques for GFPs, and the necessity of achieving optimal PBS and favorable surface topography, this study aimed to assess the surface topography and PBS of GFPs treated by sandblasting, 20% H₂O₂, and Er,Cr:YSGG laser to root dentin following cementation by precuring and co-curing methods.

## Materials and Methods

This in vitro study was conducted on 80 single-rooted human premolars extracted as part of orthodontic treatment. The study protocol was approved by the ethics committee of the university (IR.AJUMS.REC.1402.442).

### Sample size

The sample size was calculated according to a previous study [19] assuming alpha=0.05 and beta=0.2, mean bond strength values of 50.36 and 53.40 and standard deviation values of 6.67 and 7.00 in the two groups.

### Eligibility criteria

The inclusion criteria were extracted single-rooted human premolars with no caries, root length > 15 mm, no root caries, and no internal/external root resorption. Teeth with previous endodontic treatment and severe root curvature as measured by the Schneider’s method [21] were excluded.

### Specimen preparation

The teeth were decoronated by using a diamond disc under water coolant at 15 mm distance from the root apex. The root canal space was instrumented with nickel-titanium rotary files from S1 to F4 (ProTaper Universal, Dentsply Sirona, Konstanz, Germany), and irrigated with 3% NaOCl. The root canals were then obturated with 2% gutta-percha and AH26 sealer (DeTrey, Konstanz, Germany) by the lateral compaction technique. A temporary restorative material (Caviton; GC Dental Products Crop., Tokyo, Japan) was used to seal the root canal orifice. The teeth were then incubated at 37°C and 100% humidity for 1 week to ensure complete polymerization of the sealer [22]. The post space was then prepared by flaring the canal to 10 mm depth using #2 and #3 peeso reamers (Mani, Japan). The prepared post space was then irrigated with 10 mL of 3% NaOCl (to eliminate organic residues), 1 mL of ethylene diamine tetra acetic acid (MD Cleanser; Meta Biomed Co., Cheongju, South Korea) (to eliminate the smear layer), and 10 mL of distilled water, in an orderly manner [23].

Next, the teeth (n=80) were randomly assigned to four groups (n=20), and each group was randomly divided into two subgroups (n=10). The GFPs were then treated as follows:

Group 1. GFPs were sandblasted by an intraoral sandblaster (Ronving, Denmark) with 2.8 bar pressure from 10 mm distance for 20 seconds. The GFPs were then rinsed with 96% ethanol, and air-dried. Silane (Ultradent Porcelain Etch and Silane, Ultradent Products Inc, UT, USA) was then applied on the post surface for 60 seconds, dried with gentle air spray, and allowed 60 seconds before cementation.

Group 2. GFPs were immersed in 20% H2O2 at room temperature for 20 minutes. After 2 minutes of water irrigation, they were air-dried and silanized for 60 seconds. Subsequently, they were dried with gentle air spray and cemented after 60 seconds.

Group 3. GFPs were subjected to Er,Cr:YSGG laser (LightWalker STE-E Fotona Medical Lasers, Slovenia) irradiation with 2780 nm wavelength, 90 mJ energy, 4.5 W power, 50 Hz frequency, and 100 µs pulse duration for 60 seconds. Laser was irradiated at 1 mm distance with a 45-degree angle relative to the fiber post surface under water coolant. The specimens were then rinsed and air-dried. Cementation was performed after 60 seconds.

Group 4. This group served as the control group and did not receive any surface treatment. It was only silanized for 60 seconds, dried with gentle air spray, and cemented after 60 seconds. Table-1 presents the materials used in this study.

### Cementation

After post space preparation and surface treatment of GFPs, they were cemented in the two subgroups as follows:

Subgroup 1: SE precuring: After rinsing and drying of root dentin, Single Bond Universal (3M ESPE, St. Paul, MN, USA) was applied into the canal by a microbrush and air-thinned. It was then light-cured by a LED curing unit (Ivoclar, Vivadent Amherst, NY, USA) in standard mode for 20 seconds at 1 mm distance from the top as instructed by the manufacturer. The light intensity was 1250 mW/cm2 and the wavelength was 430-480 nm, confirmed by a LED radiometer before each curing cycle. Next, Rely X Ultimate Clicker cement (3M ESPE, St. Paul, MN, USA) was injected into the canal space by a mixing tip (Garant Mixing Tips Yellow, 3M ESPE, St. Paul, MN, USA). GFP was then inserted into the canal, and light curing was performed for 20 seconds.

Subgroup 2: SE co-curing: The process was the same as that in subgroup 1 except that light-curing of the bonding agent was not performed before the application of cement. Light curing was only performed once for 20 seconds after the application of cement and placement of post in the root canal system [24].

All specimens were then incubated at 37°C and 100% humidity for 24 hours prior to the PBS test.

### PBS test

**Figure-1:**
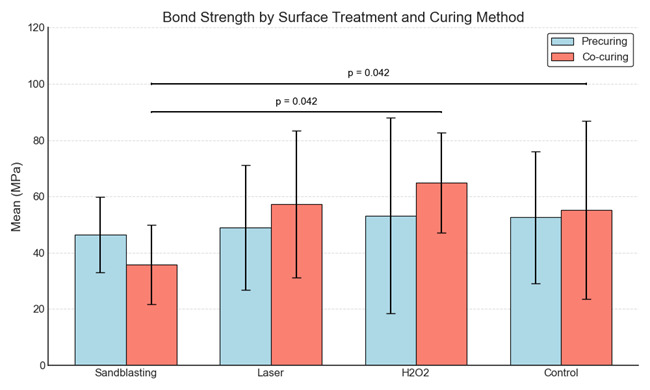
Figure 1. Mean MPa of different surface treatments under precuring and co-curing conditions. Error bars represent standard deviation. Significant differences (p < 0.05) among co-curing subgroups are indicated.

The teeth were sectioned into 1 mm thick slices by a low-speed diamond saw (WB-0060LC; PACE Technologies, Tucson, AZ, USA) under water spray. The PBS of GFPs to root dentin was then measured in a universal testing machine (JSV-H1000; JISC, Kanagawa, Japan). For this purpose, load was applied apico-cervically by a stainless-steel cylinder with a diameter matching the canal diameter at a crosshead speed of 0.5 mm/minute. The maximum force causing debonding of the post was recorded in Newtons (N). To report the PBS in megapascals (MPa), the load in Newtons was divided by the debonded surface area in square millimeters (mm2). The following formula was used to calculate the debonded surface area: A=2πrh where r is the post radius and h is the thickness of the root slice [25] (Figure-1).

### Assessment of surface topography

A total of 20 GFPs in four groups of sandblasting with 50 µm aluminum oxide particles, Er,Cr:YSGG laser, 20% H2O2, and control were inspected under a field-emission SEM (TESCAN MIRA 4, Czechia Republic) at x300 and x1000 magnifications to assess their surface topography before and after surface treatments.

### Statistical analysis

The normality of data distribution was evaluated by the Kolmogorov-Smirnov test. Accordingly, comparisons were made by one-way ANOVA, and pairwise comparisons were performed by the Tukey and Dunnett tests. The precuring and co-curing groups were compared by the Mann-Whitney test. Data were analyzed using IBM SPSS Statistics for Windows, version 27 (IBM Corp., Armonk, N.Y., USA) at 0.05 level of significance.

## Results

**Figure-2:**
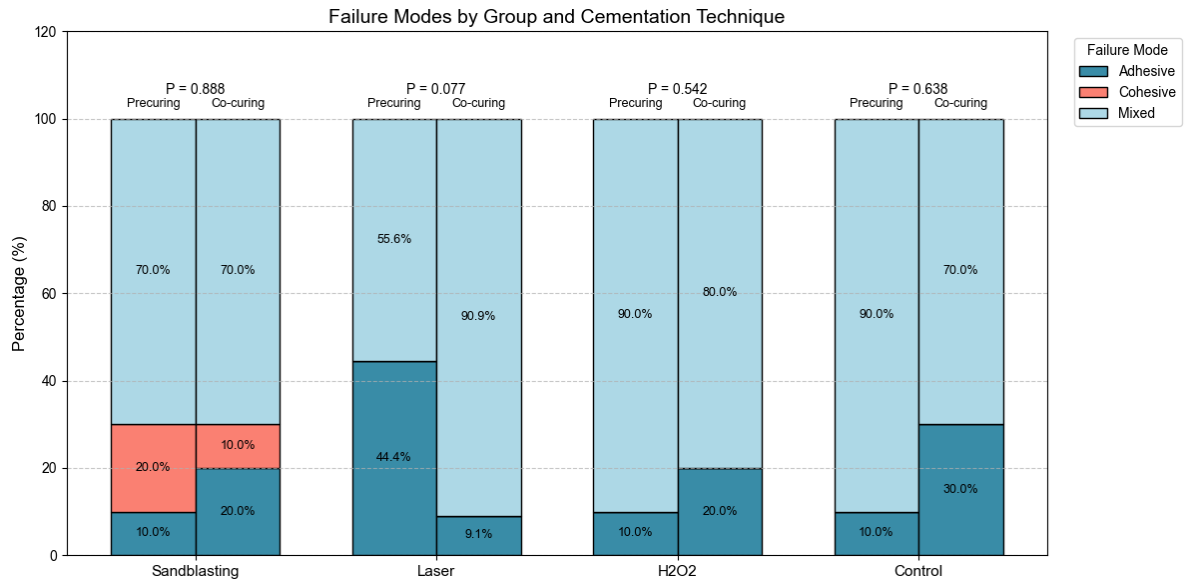
Figure 2. Mode of failure in the four groups divided by the precuring or co-curing status; p values show within group comparisons of precuring or co-curing groups in each surface treatment types.

**Figure-3:**
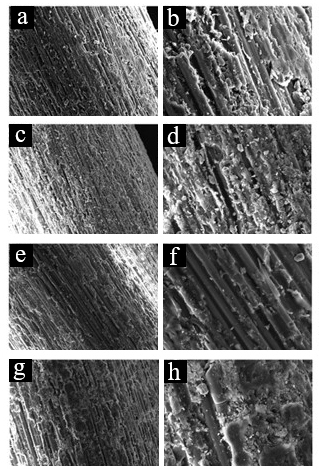
Figure 3. SEM images of GFP surfaces (×1000 and ×300 magnification, respectively) for the control (a, b), H₂O₂-treated (c, d), laser-treated (e, f), and sandblasted (g, h) groups.

**Table:** Table1. Materials used in this study

Material	Manufacturer	Composition
		Base paste: Methacrylate monomers, silanted fillers, initiator
Rely X Ultimate Clicker	3M-ESPE	components Catalyst Paste: Methacrylate monomers, alkaline (basic) fillers, initiator components
Single Bond Universal Adhesive	3M- ESPE	MDP, Phosphate Monomers, Dimethacrylate resins, HEMA, Vitrebond Copolymer, Filler, Ethanol, Water, Silane, Initiators
Glassix Fiber Post #2	NORDIN	Epoxy resin and glass fiber, braided plait in multi-axial arrangement

**Table:** Table2. Mode of failure based on the cementation technique

Subgroup		Frequency	Percentage	p-value
	Adhesive	7	17.9	
Precuring	Cohesive	2	5.1	
	Mixed	30	76.9	
	Total	39	100.0	0.952
	Adhesive	8	19.5	
Co-curing	Cohesive	1	2.4	
	Mixed	32	78.0	
	Total	41	100.0	

### PBS

The mean PBS values in MPa were as follows: Sandblasting precuring (n=10) showed 46.48 ± 13.45 (range 20.8-64.5), while co-curing (n=10) was lower at 35.88 ± 14.10 (range 15.4-51.2). Laser precuring (n=9) was 49.02 ± 22.22 (range 25.3-90.2), co-curing (n=11) was higher at 57.25 ± 26.05 (range 21.3-105.0). H₂O₂ precuring (n=10) was 53.23 ± 34.80 (range 25.6-121.0), co-curing (n=10) was 64.90 ± 17.77 (range 30.5-94.3). Control precuring (n=10) was 52.60 ± 23.49 (range 11.0-89.4), co-curing (n=10) was 55.21 ± 31.68 (range 10.7-118.0). Among the co-curing subgroups, pairwise comparisons (Tukey HSD) showed significant differences only between Sandblasting co-curing and Control co-curing (mean difference 19.33 MPa, p=0.042) and between Sandblasting co-curing and H₂O₂ co-curing (mean difference -29.02 MPa,

### Mode of failure

As shown in Figure-2, mixed failures were the most frequent across all groups. In the Sandblasting group, mixed failures occurred in 70% of both Precuring and Co-curing subgroups (adhesive 10-20%, cohesive 10-20%; P = 0.888). In the Laser group, mixed failures were 55.6% in Precuring and 90.9% in Co-curing (adhesive 9.1-44.4%; P = 0.077). For H₂O₂, mixed failures accounted for 90% and 80% in Precuring and Co-curing, respectively (adhesive 10-20%; P = 0.542). In the Control group, mixed failures were 90% in Precuring and 70% in Co-curing (adhesive 10-30%; P = 0.638).

Also, another comparison was made considering control group as reference. Comparison of each intervention group with the control group regarding the mode of failure revealed no significant difference between the sandblasting and control groups (P=0.330 for precuring and P=0.888 for co-curing method), laser and control (P=0.098 for precuring and P=0.234 for co-curing), and H2O2 and control (P=1.00 for precuring and P=0.615 for co-curing method) groups (Table-2).

Irrespective of surface treatments, in both pre-curing and co-curing cementation techniques, the majority of failures were of mixed type, accounting for 76.9% and 78.0%, respectively. Adhesive failures were observed in 17.9% of pre-curing and 19.5% of co-curing cases, while cohesive failures were the least frequent in both groups.

### Surface topography

Figures 3 shows the surface topography of GFPs in the four groups. In the control group (Figure 3.a.b), GFPs were coated with epoxy resin almost completely. As shown in Figure 3.c.d, etching with H2O2 increased the surface roughness along the post, and fibers had no fracture. In the laser group (Figure 3.e.f), diluted resin was seen in the matrix around the fibers, and fibers were free from resin. Also, micro-cracks and grooves were seen, which could have increased the bond strength. As shown in Figure 3.g.h, sandblasting yielded a rougher surface than the control group and some fractures were noted in the fibers.

## Discussion

This study assessed the surface topography and PBS of GFPs treated by sandblasting, 20% H₂O₂, and Er,Cr:YSGG laser to root dentin following cementation by precuring and co-curing methods. The results showed that in H₂O₂-treated GFPs, the mean PBS was significantly higher in the co-curing than pre-curing method. Conversely, no significant difference was found between the precuring and co-curing methods in PBS of GFPs with other surface treatments. Regarding cementation with the precuring method, the PBS of laser, sandblasting, and H₂O₂ groups was not significantly different. However, in the co-curing method, sandblasting significantly decreased the PBS but surface treatment with laser and H₂O₂ had no significant effect on PBS.

The fiber post matrix is composed of epoxy resins, which do not have any functional group for reaction with resin cement monomers, a characteristic that results in a lower bond strength. Accordingly, surface treatment of fiber posts is recommended to change the surface energy level and enhance its wettability by creating a rough surface and exposure of fibers to increase the available surface area for chemical bonding [26][27]. Moreover, the gap between the fibers may provide an additional space for micro-mechanical retention of resin, thereby increasing the final PBS of fiber posts [27]. Silanization, application of H₂O₂ or methylene chloride, hydrofluoric acid etching, air abrasion, and laser irradiation are among the most commonly used surface treatments for fiber posts [27][28].

The present results regarding no significant effect of cementation technique (precuring/co-curing) on PBS of GFPs to root dentin was in agreement with the results of Chou et al, [19] who assessed the PBS of GFPs cemented with SE bonding systems with precuring and co-curing methods, and found no significant difference in PBS between the tested methods.

In the current study, surface treatment of GFPs with Er,Cr:YSGG laser (2780 nm, 90 mJ, 4.5 W, 50 Hz, for 60 seconds) had no significant effect on the PBS of GFPs to root dentin, compared with the control group. The same results were reported by Davoudi et al, [27] in their systematic review. Similarly, Alonaizan et al. [29] reported that surface treatment of fiber posts with Er,Cr:YSGG laser had no significant effect on their PBS.

Unlike the present results, Mekky et al. [5] reported an increase in PBS of GFPs treated with Er,Cr:YSGG laser (2790 nm, 150 mJ, 1.5 W, 10 Hz frequency, 60 seconds) compared with the control group. It is important to note that length and surface topography of fiber posts are important determinants of a strong bond. Also, the PBS is influenced by the type of fiber post and laser exposure parameters [27].

The present results indicated that sandblasting with 50 µm aluminum oxide particles with 2.8 bar pressure decreased the PBS compared with the control group in the co-curing cementation method. However, it had no significant effect on the PBS in the pre-curing method. Similarly, Mekky et al. [5] reported that air abrasion with 50 µm aluminum oxide particles did not increase the PBS of fiber posts compared to the control group. Furthermore, Moghaddas and Borouziniat [30] demonstrated that air abrasion by sandblasting damaged the fibers on the surface of fiber posts, and had no significant efficacy for improvement of the pull-out bond strength of quartz fiber posts. Their results were in line with the present findings. In contrast, Davari et al. [31] showed that sandblasting with 30 µm silica-coated aluminum oxide particles with 2.8 bar pressure by Cojet increased the PBS of QP fiber post, compared with the control group. Additionally, Karunakaran et al. [28] reported that sandblasting with 110 µm aluminum oxide particles increased the PBS of quartz and GFPs compared to the control group. Malekipour Esfahani et al. [32] found that surface treatment of quartz fiber posts with air abrasion (110 µm aluminum oxide particles with 2.8 bar pressure for 60 seconds) increased the PBS. These discrepancies suggest that variations in particle size, duration of sandblasting, and distance between the posts and tip of the device in the two studies may explain the differences in the results.

The present results indicated that surface treatment of GFPs with 20% H₂O₂ had no significant effect on their PBS. Unlike the present results, Malekipour Esfahani et al. [32] demonstrated that surface treatment of quartz fiber posts with 10% H₂O₂ for 20 minutes increased their PBS. The difference between their results and the present findings may be due to using a different type of post and a difference in concentration of H₂O₂.

Mixed failure was dominant in all groups in the present study, followed by adhesive failure. Adhesive failure is clinically more favorable than cohesive failure because repair of a tooth with a broken post is only possible by complete retrieval of the post [27]. It should be noted that bonding to root dentin is challenging due to its complex anatomy, and difficult control of the adhesive system. The C factor is highly important in bonding to root dentin, since it can increase the polymerization stress of resin cements, and decrease the surface energy of root dentin; thus, some gaps may develop at the adhesive interface and compromise the durability of restoration [28].

## Strengths and limitations of study

Assessment of PBS was a strength of the present study since it provides more accurate information than the conventional shear bond strength tests, and failure parallel to the cemented post-dentin interface better simulates the clinical setting [28][31]. However, the in vitro design was a limitation of this study, which prevents complete generalization of the results to the clinical setting.

## Conclusion

In H2O2-treated fiber posts, the co-curing cementation method yielded a significantly higher PBS than the pre-curing method. Cementation technique had no significant effect on fiber posts with other surface treatments. In pre-curing cementation method, the tested surface treatments had no significant effect on PBS compared with the control group; however, in the co-curing method, sandblasting significantly decreased the PBS while other surface treatments had no significant effect on PBS of GFPs to root dentin.

## Conflict of Interest

The authors declare no conflict of interest.

## AI Disclosure Statement

During the preparation of this manuscript, the authors used ChatGPT, OpenAI company for language editing, grammar improvement, and liboberry.com for reference management. After its use, the authors thoroughly reviewed, verified, and revised all AI-assisted content to ensure accuracy and originality. The authors take full responsibility for the integrity and final content of the published article.
